# Cellular Homeostasis and Antioxidant Response in Epithelial HT29 Cells on Titania Nanotube Arrays Surface

**DOI:** 10.1155/2017/3708048

**Published:** 2017-02-28

**Authors:** Rabiatul Basria SMN Mydin, Srimala Sreekantan, Roshasnorlyza Hazan, Mustafa Fadzil Farid Wajidi, Ishak Mat

**Affiliations:** ^1^Advanced Medical and Dental Institute, Universiti Sains Malaysia, Bertam, 13200 Kepala Batas, Penang, Malaysia; ^2^School of Materials and Mineral Resources Engineering, Universiti Sains Malaysia, Engineering Campus, Nibong Tebal, 14300 South Seberang Perai, Penang, Malaysia; ^3^Materials Technology Group, Industrial Technology Division, Nuclear Malaysia Agency, Bangi, 43000 Kajang, Selangor, Malaysia; ^4^School of Distance Education, Universiti Sains Malaysia, 11800 Penang, Malaysia

## Abstract

Cell growth and proliferative activities on titania nanotube arrays (TNA) have raised alerts on genotoxicity risk. Present toxicogenomic approach focused on epithelial HT29 cells with TNA surface. Fledgling cell-TNA interaction has triggered G0/G1 cell cycle arrests and initiates DNA damage surveillance checkpoint, which possibly indicated the cellular stress stimuli. A profound gene regulation was observed to be involved in cellular growth and survival signals such as p53 and AKT expressions. Interestingly, the activation of redox regulator pathways (antioxidant defense) was observed through the cascade interactions of GADD45, MYC, CHECK1, and ATR genes. These mechanisms furnish to protect DNA during cellular division from an oxidative challenge, set in motion with XRRC5 and RAD50 genes for DNA damage and repair activities. The cell fate decision on TNA-nanoenvironment has been reported to possibly regulate proliferative activities via expression of p27 and BCL2 tumor suppressor proteins, cogent with SKP2 and BCL2 oncogenic proteins suppression. Findings suggested that epithelial HT29 cells on the surface of TNA may have a positive regulation via cell-homeostasis mechanisms: a careful circadian orchestration between cell proliferation, survival, and death. This nanomolecular knowledge could be beneficial for advanced medical applications such as in nanomedicine and nanotherapeutics.

## 1. Introduction

Nanotechnology has become a foremost research interest as this technology provides opportunity to explore functional devices at nanometer regime and makes it possible to design materials compatible with nanoscale topography. Titania nanotube array (TNA), also referred to as titanium dioxide (TiO_2_) nanotube array, has been proposed as a new interactive alternative to the existing human implant material surface as the former possesses better cytocompatibility and osseointegration responses [1–4]. The unique structure of TNA promotes an increased surface area of three times creating more space for cellular interaction [5–8]. Furthermore, this nanoscale surface can also pile up surface energy for greater adhesion protein selection such as vitronectin and fibronectin; thus these factors accord to the cell growth, proliferation, and differentiation activities [9–13]. However, uncontrolled cell activities are at high risk for genetic instability and carcinogenesis.

Furthermore, the emergence of nanotechnology in producing nanosized material has instigated many concerns about the effect of nanoparticles on human health, toxicity of the material, and safety of the environment. Nanomaterials are highly reactive even if inert metals like gold are used; this is due to the nanodimensions properties [14–16] as they are able to generate reactive oxygen species (ROS) or reactive nitrogen species (RNS) [17]. Both ROS and RNS have vital functions in regulating cellular metabolism, proliferation, gene expression, and signal transduction involved in numerous physiological activities (e.g., inflammation, ventilation, and blood pressure control). Untrammeled ROS production has been associated with chronic oxidative stress [18] and could become toxic to cells which usually intricate the DNA damaged activity [19–21]. Moreover, several studies have linked TiO_2_ nanoparticles and ROS activities to diseases and cancer development [22–26]. Therefore, recently a key concern has raised whether TNA could lead to these harmful risks.

From the molecular biology point of view, the synergistic effect of nanomaterial's potential hazard to cell proliferation response can be monitored by investigating various gene and protein associated with cell cycle checkpoint or circadian regulator [27], DNA damage surveillance, and DNA repair mechanism. The circadian protein is a cell cycle regulator responsible for cell growth and proliferation activities will be influenced by stress stimuli from ROS and oxidative stress resulted from cell-nanomaterial interactions [28–32]. The cell might determine its fate either by activation of survival pathways to increase protective or destructive stress reactions which could initially involve the DNA damage surveillance and repair mechanism [33].

Beyond that, circadian oscillator is vital for an antioxidant defense mechanism possibly via cell-homeostasis response in order to protect macromolecules of the cells especially the DNA from oxidative challenge during cellular division [34–37]. Homeostatic proliferation is the orchestrated changes in cellular metabolism to support the cell physiological state. Therefore, the aim of our study was to investigate detail on cell-TNA response focusing on cell cycle profile, DNA damage, DNA repair, oncogene, or tumor suppressor activities. This study will contribute to the knowledge on nanomolecular aspect of TNA to develop better nanomaterials for biomedical and nanomedicine applications.

## 2. Materials and Methods

### 2.1. Study Material

Titanium foil (0.13 mm thickness, 99.6% purity, STREM Chemicals) was cut into small pieces of 5 cm × 2 cm. The pieces were ultrasonically cleaned with acetone and subsequently rinsed in deionized water. The sample was then anodized in glycerol (85%, MERCK) containing 5wt% ammonium fluoride (MERCK, 87%) with DC power supply at 30 V for 30 mins. Fresh electrolyte was used for TNA formation. After anodization process, the sample was rinsed with deionized water and dried in air. The anodized titanium was annealed at 400°C in argon for 2 h in order to obtain a crystalline phase, anatase. Field emission scanning electron microscope (FESEM) and energy dispersive X-ray spectroscopy (EDX) were used to characterize the TNA surface. The test material surface was TNA, while raw titanium foil (TiP) and glass surface (GS-Thermo Scientific Nunc) were selected as control materials. The glass slides were cleaned with 7X detergent, rinsed overnight under running tap water, and finally soaked in 70% ethanol and air-dried. The dimensions of each test material were standardized at 2 cm × 2 cm and quadrupled for each test. The materials were stored in vacuum drying oven at 45°C for 24 h to remove air trapped and pretreated with cell growth medium before proceeding to the cellular study. This action could minimize the cell interfacial stress and delamination factors due to limited growth nutrients for cells.

### 2.2. Cell Culture

The epithelial HT29 cells (ATCC HTB 38) were obtained from the American Type Culture Collection (Manassas, VA) and maintained in fresh culture medium containing RPMI 1640 medium, 10% fetal bovine serum (FBS), 5% L-glutamine, and 5% penicillin in a humidified incubator with an atmosphere of 5% CO2 + 95% air at 37°C. All the test materials were placed in 6-well cell culture plate (Corning-Costar) and seeded with the HT29 cells at a density of 1 × 10^5^ cell per well in 4 mL culture medium. The cultured plates were maintained for one week with daily observation until 90% cell confluency was reached. Alternately, in every 72 hours, the new culture medium was replaced in each well. The cells were grown on each surface such that TNA, TiP, and GS were analyzed. Additionally, cells grown on ordinary culture system surface (plastic), represented as a control, were also analyzed, whereas cell culture plate surface treated nicotine (NQ) is represented as positive control for cellular stress, oxidative stress, and genotoxicity risk for the cells [38].

### 2.3. DNA Cell Cycle Pattern

Commercially available CycleTest-Plus DNA reagent kit (Becton Dickinson, Mount View, CA) was used to stain the cells by propidium iodide. Cell suspensions from other control cultures or treated cultures were collected by trypsinization and washed twice with buffer solution (provided). The cells were counted and adjusted to a concentration of 1.0 × 10^6^ cells/mL. The nuclei were labelled with propidium iodide according to manufacturer's protocol. Fluorescence data related to the DNA content of the cells in different cell cycles were collected with the Becton Dickinson FACSCalibur flow cytometer. Assays were performed at least three times, and data shown were representative of these assays. The data were analyzed using ModFit LT 3.3 software (Verity Software House, Inc., ME).

### 2.4. Gene Expression

Total RNA samples were extracted using the RNeasy mini kit (Qiagen) and measured with nanospectrophotometer for 260/280 ratio (1.9–2.1) and 260/230 ratio (>1.5). The complementary DNA (cDNA) was prepared using High Capacity RNA-to-CDNA Kit (Applied Biosystems) following the manufacturer's protocol. The real-time polymerase chain reaction (RT-PCR) assays were performed using TaqMan® Array 96-Well Fast Plates that contained triplicate sets of primers for manufacturing control (18S rRNA), housekeeping genes (EGFR and HNRNPA2B1), and genes of interest (Table 1). The plates containing each sample were then loaded into the thermal cycler StepOnePlus™ systems by using the thermal profile of 95°C 20 s/[95°C  3 s − 60°C  30 s] × 40. The data obtained from the gene expression assay were analyzed by comparative cycle threshold method and relative expression of the housekeeping genes. Further statistical analysis was performed using one-way analysis of variance (ANOVA) with the help of SPSS software version 22.

### 2.5. Protein Expression

The protein samples with 20 *μ*g concentration per sample were prepared using cell extraction buffer (Invitrogen, FNN0011) and separated on 10% sodium dodecyl sulfate (SDS) polyacrylamide gels, then electrophoretically transferred (200 mA, 2 h) to a polyvinylidene difluoride (PVDF) membrane (0.45 *μ*m pore size, Millipore) before proceeding with immunoblot detection. Antibodies were from the following: BCL2-Monoclonal Mouse (Invitrogen, 1224968A), p27-Polyclonal Rabbit (Cell Signaling, 2552), pRB-Mouse Monoclonal (BD Pharmingen, 554136), and SKP2-Polyclonal Rabbit (Cell Signaling, 4358). Beta-Actin-Polyclonal Rabbit (Cell Signaling, 4967S) was used as a reference signal.

## 3. Results

**Figure 1:**
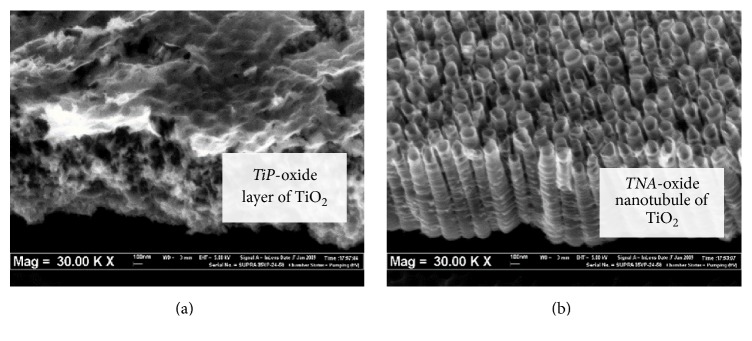
Field emission scanning electron microscopy image for (a) TiP and (b) TNA surface. The anodization process on TiP contributed to the formation of nanotubular oxide structure of TiO_2_, denoted as titania nanotubes arrays.

**Figure 2:**
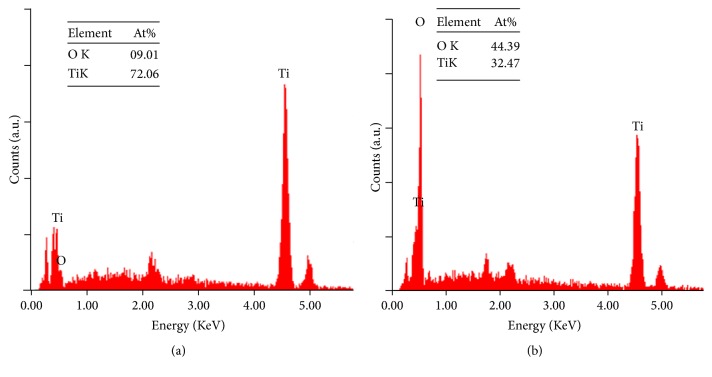
EDX profiles for (a) TiP and (b) TNA. The formation of TiO_2_ nanotubes on TNA surface probably contributed to the presence of a higher oxide element on TNA compared to TiP surface.

Originally, the surface of titanium metal (TiP) was observed to have a grainy TiO_2_ layer. The surface modification by anodization on TiP produced a nanotubular structure of TiO_2_, denoted as TNA (Figure 1). The formation of well-aligned nanotubular structure (nanotubes) was observed to have the following measurements (average): outer diameter: 100 nm; inner diameter: 60 nm; wall thickness: 15 nm; and length: 600 nm. EDX element surface analysis profile of TNA and TiP material surfaces clearly showed the presence of titanium and oxygen element (Figure 2). The oxygen content was found to be higher compared to the TiP due to the thick layer of TiO_2_ nanotubular structure on flat titanium surface. EDX element analysis of TiP showed it was rich with Ti element originating from Ti substrate. The presence of oxygen on TiP indicated a thin layer of oxide formed on the Ti substrate, which probably resulted from an atmospheric oxidation process.

**Figure 3:**
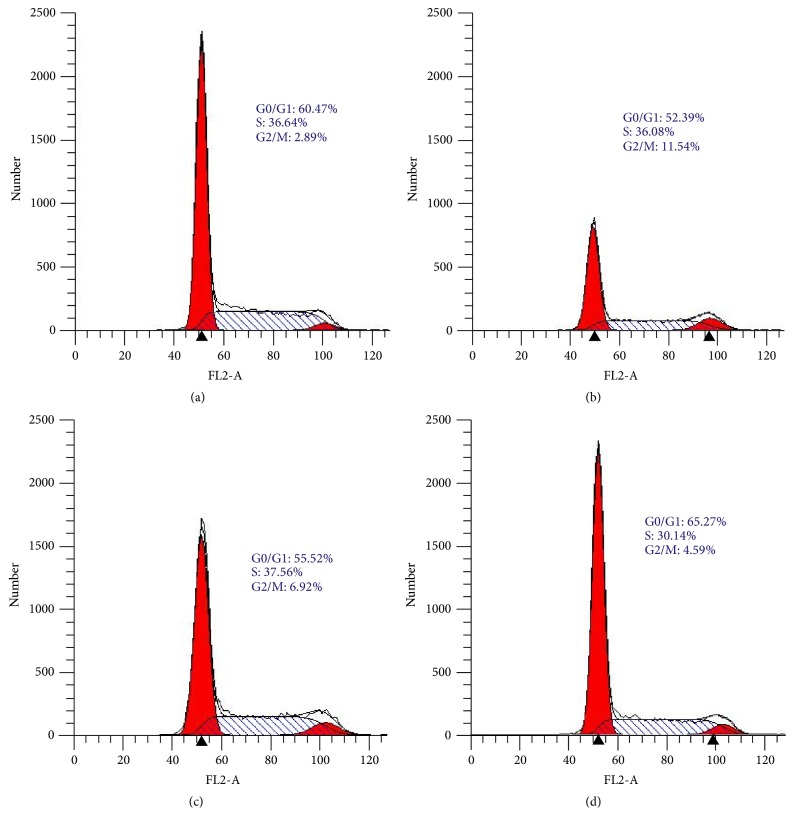
Effect of TNA surface on cell cycle progression at 7-day culture. The epithelial HT29 cells were cultured on (a) plastic, (b) glass, (c) TiP, and (d) TNA surfaces. Cell cycle profile from ModFit LT software represented the percentage of cell cycle distribution that could be divided into three phases, namely, G0/G1 phase, S-phase, and G2/M phase. Results were obtained from two independent experiments, and one representative result is presented.

The effect of TNA nanosurface on cell cycle progression for a 7-day incubation period was studied on epithelial HT29 cells, compared to control surfaces, namely, plastic, glass, and TiP. Along the period (Figure 3), TNA nanosurface caused an increased cell growth arrest at G0/G1 (65.27%) compared to plastic (60.47%), glass (52.39%), and TiP (55.52%). The cell growth arrest at G0/G1 was followed by the reduction in S-phase with 30.14% for TNA surface, compared to plastic (36.64%), glass (36.08%), and TiP (37.56%). The G2/M phase cell cycle distribution slightly increased on TNA surface (4.59%) compared to plastic (2.89%). However, the percentage of cells for TNA at G2/M phase was lower compared to glass (11.54%) and TiP (6.92%). Cell-TNA interaction at 7-day culture regulates the cell cycle progression via G0/G1 phase arrest.

**Figure 4:**
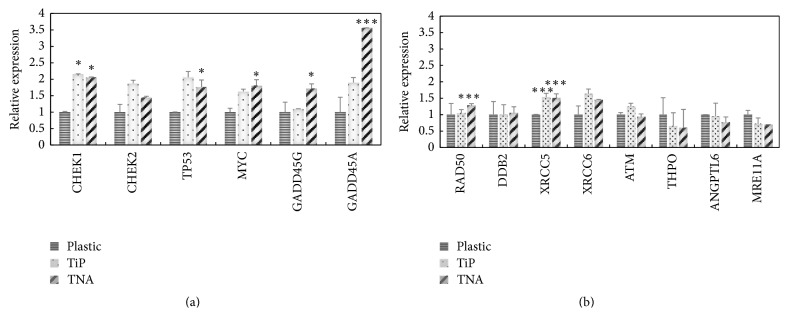
Gene expression profile of genes involved in (a) cell cycle arrest mechanism and (b) DNA repair mechanism. The study material surfaces were TNA (N), TiP (T), and plastic (P). Data points represented means ± SEM of triplicate observations from a representative experiment, ^*∗*^*p* < 0.05 (significant) and ^*∗∗∗*^*p* < 0.001 (extremely significant) based on one-way ANOVA.

The regulation of cell cycle arrest on TNA surface was further investigated involving multiple genes, namely, CHEK1, CHEK2, TP53, MYC, GADD45G, and GADD45A, at mRNA expressions level (Figure 4(a)). Epithelial HT29 gene expression profile showed increasing patterns of CHEK1 (*p* < 0.05), CHEK2, TP53 (*p* < 0.05), MYC (*p* < 0.05), GADD45G (*p* < 0.05), and GADD45A (*p* < 0.001) genes for TNA surface compared to their pattern on plastic surface. The expression of CHEK1, CHEK2, and TP53 genes on TNA surface was slightly reduced compared to their expression on TiP surface. These findings suggest that cell-TNA interaction could involve the cell cycle arrest mechanism.

In order to obtain a thorough understanding on DNA repair mechanism on cell-TNA interaction, important genes, namely, RAD50, DDB2, XRCC5, XRCC6, ATM, THP0, ANGPTL6, and MRE11A, were further profiled at mRNA expression level on epithelial HT29 cells compared to control surfaces, namely, plastic, glass, and TiP (Figure 4(b)). Increasing expression patterns of RAD50 (*p* < 0.001) and XRCC5 (*p* < 0.001) genes on TNA surface were observed compared to control surfaces. XRCC6 gene expression showed an increasing pattern on TNA surface compared to plastic surface. DDB2 and ATM genes showed no significant difference between all the material surfaces. THPO, ANGPTL6, and MRE11A genes showed decreased expressions on TNA surface compared to control surfaces. These findings suggest that cell-TNA interaction might be involved in DNA repair mechanisms via the activation of XRCC5 and RAD50 activities.

**Figure 5:**
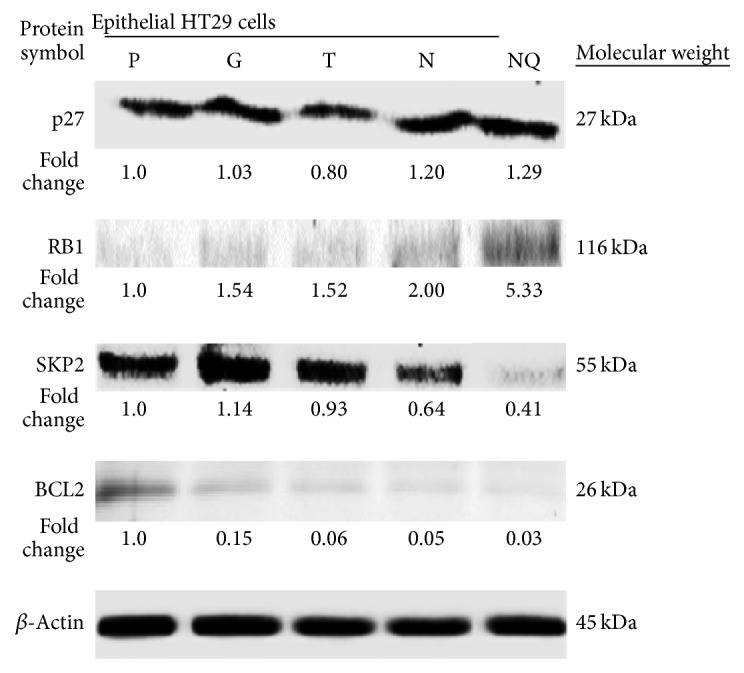
Immunoblot analysis of selected tumor suppressor and oncogene proteins. Expression of tumor suppressor proteins (p27 and RB1) and suppression of oncogene proteins (SKP2 and BCL2) were observed in epithelial HT29 cells grown on TNA surface. *β*-Actin protein profile was used as a loading control. The studied protein samples were obtained from TNA (N), TiP (T), glass (G), plastic (P), and nicotine-treated surface (NQ). Results were obtained from two independent experiments, and one representative result is presented.

The cell-TNA involvement in oncogene mechanism was studied using protein markers, namely, SKP2 and BCL2, on epithelial HT29 grown on different material surfaces. The protein expression of SKP2 was detected using immunoblotting at 55 kDa molecular weight (Figure 5). The TNA surface showed a decreased SKP2 protein expression at 0.64-fold change compared to control surfaces, namely, plastic (1-fold change), glass (1.41-fold change), and TiP (0.93-fold change). The cells with NQ treatment showed a much decreased SKP2 expression at 0.41-fold change. Next, the expression of BCL2 protein was detected at 26 kDa molecular weight. Inhibition of BCL2 expression on TNA surface was observed at 0.05-fold change in comparison to control surfaces, namely, plastic (1-fold change), glass (0.15-fold change), and TiP (0.06-fold change). The cells on control surface with NQ treatment showed an inhibition of BCL2 at 0.33-fold change. Results from this study indicate the activation of tumor suppressor proteins such as p27 and RB1 coherently triggered with suppressor oncogene proteins such as SKP2 and BLC2.

The cell-TNA involvement in oncogene mechanism was studied using protein markers, namely, SKP2 and BCL2, on epithelial HT29 grown on different material surfaces. The protein expression of SKP2 was detected using immunoblotting at 55 kDa molecular weight (Figure 5). The TNA surface showed a decreased SKP2 protein expression at 0.64-fold change compared to control surfaces, namely, plastic (1-fold change), glass (1.41-fold change), and TiP (0.93-fold change). The cells with NQ treatment showed a much decreased SKP2 expression at 0.41-fold change. Next, the expression of BCL2 protein was detected at 26 kDa molecular weight. Inhibition of BCL2 expression on TNA surface was observed at 0.05-fold change in comparison to control surfaces, namely, plastic (1-fold change), glass (0.15-fold change), and TiP (0.06-fold change). The cells on control surface with NQ treatment showed an inhibition of BCL2 at 0.33-fold change. Results from this study indicates the activation of tumor suppressor proteins such as p27 and RB1 coherently triggered with suppressor oncogene proteins such as SKP2 and BLC2

## 4. Discussion

Field emission scanning electron microscope (FESEM) analysis on TNA nanoarchitecture showed the presence of the hole-like structure and gaps between TiO_2_ nanotubular structure, which could be beneficial as a supply or storage route for growth nutrients and mediator growth signals. These signals are essential biological component for cell growth activities such as cell adhesion, migration, proliferation, and differentiation [39, 40]. Several studies have reported that TNA nanoscale surfaces enhance cell activities on various types of cells such as epithelium, fibroblast, and osteoblast [41–44]. Furthermore, the TNA nanostructure consists of a higher oxide layer of TiO_2_ as a result from the larger surface area. Surface topography and chemical composition of the TNA play a vital role for the cell morphology and behavior responses. However, a recent study shows that the nanomaterial's physicochemical properties also play an important role to control the risks arising from its cytotoxicity, genotoxicity, and even carcinogenicity [45].

In this study, cellular response on TNA nanosurface was found to be involved in G0/G1 cell cycle arrest (Figure 3) at an incubation period of 7 days for epithelial HT29 cells. The G0/G1 arrest might trigger an involvement of various cell cycle regulatory proteins (checkpoint proteins) such as CDKs and kinases. As such, this checkpoint would be a control for the cell size, nutrients, growth factors, and DNA damage [46]. Moreover, the “checkpoints” would be cued from cells, responding to either entry or exit from the distinct cell cycle phases. Furthermore, the cellular stress from cell-TNA adaptation responses triggers cell cycle arrest which possibly contribute to the cellular improvement activity towards the nanosurface interactions.

The expression of DNA-damage-inducible protein (GADD45) has been reported to act as a stress sensor towards the stressors and may involve cell cycle arrest activity [47]. The findings from this study indicated significant increases of GADD45A and GADD45G genes expression on cell-TNA interaction in epithelial HT29 cells (Figure 4(a)). Therefore, these findings suggested that TNA nanosurface interaction could possibly act as a stress agent (stressor) that results in cytochemical stress. Furthermore, cell-TNA stimulus may regulate the circadian rhythm activity in monitoring cell cycle progression via the DNA damage surveillance mechanism. This study revealed that gene profile was involved in the DNA damage surveillance mechanisms such as checkpoint kinase I (CHEK1), checkpoint kinase II (CHEK2), tumor protein p53 (TP53), and V-MYC myelocytomatosis viral oncogene homolog (MYC). Phosphorylation of CHEK1 and CHEK2 was responsible for serine/threonine-specific protein kinase pathways, which resulted from the activation of DNA damage sensor proteins such as the ataxia telangiectasia mutated (ATM) protein together with ataxia telangiectasia and Rad3 related (ATR) protein. The activation of ATM or ATR occurred due to an involvement of core circadian proteins, circadian protein homolog 1 (PER1), or T-cell immunoglobulin domain and mucin domain (TIM1), which served as a cofactor or adaptor protein, respectively. Later on, this interaction would signal to the cell cycle progression with potential genomic organization's activities [48].

Nevertheless, the repression level of genes such as CHEK1, CHEK2, and TP53 on TNA-cells compared to their repression level on TiP cells was observed. This was done to rescue the proliferative competent cells from tumorigenesis risk (safeguard program). TP53 expression would represent a stress-responsive gene, directing the activity of p53 protein as a tumor suppressor product [49]. Activation of p53 protein may act together with MYC protein (also known as regulator gene for transcription factor) involved in several other responses such as transcriptional genes in the cell cycle, DNA repair, and apoptosis regulations [50].

Generally, the cell expresses a normal level of p53 during a process of the cell cycle with relatively short half-life and then rapidly targets ubiquitination and degradation [51]. The gene phosphorylations may respond to the cell-TNA oxidant stress resultant from the generation of free radical and reactive oxygen species (ROS). Untrammeled oxidant stress could raise a risk in DNA damage and genotoxicity risk [52]. Thus, the expression and activation of circadian clock proteins might indicate the antioxidant defense mechanism in order to protect the DNA from oxidative challenge during cellular division [53, 54].

In addition, the mitotic delay observed earlier might also be due to an activation of another metabolic process in DNA repair and cell improvement activity [55, 56]. The damaged DNA would be sensed by “sensor” proteins and signals to “transducer” proteins and activates cascade “effector” proteins associated to the cell repair mechanisms [57]. The findings from the present study suggested that the DNA repair mechanism might be regulated through X-Ray Repair Cross-Complementing Protein 5 (XRCC5) and DNA repair protein RAD50 (Figure 4(b)) in epithelial HT29 cells. DNA repair protein XRCC5, Ku heterodimer protein (80-kDa), is referred to as ATP-dependent DNA helicase II, while RAD50 is a protein involved in DNA double-strand break (DSB) repair. The connection of XRRC5 and RAD50 expression may be involved in central role* DSB *repair, DNA recombination, and meiosis [58]. The in vitro cell line model used in this study may represent the existing damaged or altered DNA sequence due to a rapid cell proliferative characteristic. Hence, this study predicted that cell-TNA regulation might start with an activation of DNA damage sensor protein, which later on would activate a repair mechanism to improve the cellular performance. Arguably, the cell-TNA interactions might also signal the cellular senescence pathway and/or cell-homeostasis activities in order to overcome the cellular stress.

In this study, the findings indicated that tumor suppressor protein and oncoprotein interactions represent an important role in cell fate decision between cell survival and apoptosis regulation. The oncoprotein, namely, SKP2, which functions as a key regulator in the G1-S transit of the cell cycle, is often overexpressed in human cancer. Inhibition of SKP2 expression leads to growth arrest, apoptosis, and reduced cell migration in terms of invasion or metastasis [53]. It was observed in the study that cell-TNA interaction might suppress the expression of SKP2 protein, a suppression which could then contribute to the adhesion-independent ability and carcinogenesis control [59]. The expression of SKP2 may act together with p27 protein, a cell cycle regulator protein and a tumor suppressor protein. Study by Chen and Tweddle [60] reported that SKP2 protein overexpression may lead to accelerated p27 proteolysis and carcinogenesis activity. However, this study found that cell-TNA interaction may suppress the SKP2 expression and lead to an accumulation of p27 expression, which might contribute to a critical control of cell cycle progression.

Furthermore, researchers have also found that the p27 and SKP2 pathway may act as the inhibitors of tumor progression in RB function [61]. The cell cycle could be arrested through a molecular interaction of RB protein to N terminus of SKP2 that would interfere in SKP2-p27 interaction and inhibit proteolysis of p27. Besides, RB is also known as a “master brake” to the cell cycle progression at G1 checkpoint (restriction point), which is responsible for the DNA replication and proliferation [62]. It was evident from this study that the cell-TNA interaction activated the expression of RB protein to inhibit extreme (uncontrolled) cell growth by preventing cell cycle progression possibly through the E2F transcription pathway, the checkpoint on cell condition for DNA replication and DNA repair. Thus, when the cells pass through checkpoint conditions, pRB is phosphorylated and becomes inactive and eventually allows cell cycle progression [63].

**Figure 6:**
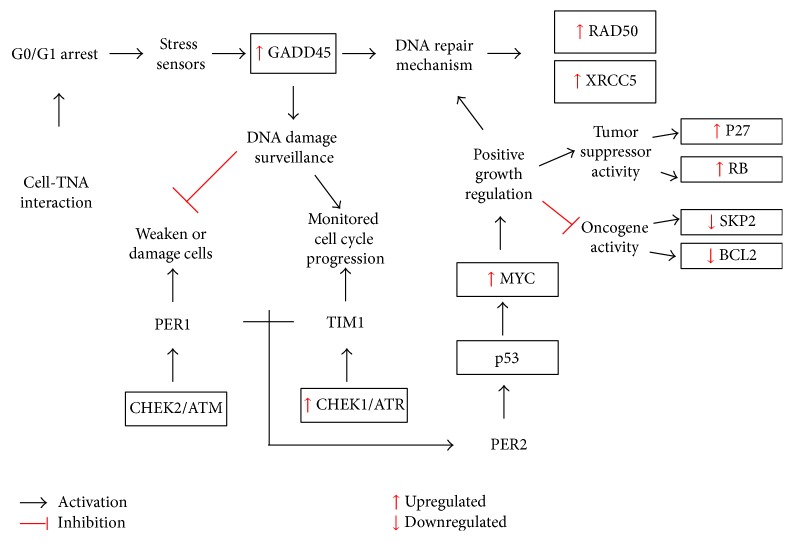
The schematic representative G0/G1 cell cycle arrest through stress sensor activity and activation of positive growth regulation on cell-TNA stimulus. This mechanism was predicted based on the epithelial HT29 cell line model.

A part from that, the apoptosis-associated protein, BCL2, was studied to gain a clearer understanding of the molecular mechanism in cell fate decisions. BCL2 is an apoptosis regulator protein that regulates cell death by either stimulating (proapoptotic) or hindering (antiapoptotic) apoptosis. Besides, BCL2 is also classified as an oncogene [64]. This study found that the cell-TNA interaction inhibited BCL2 protein expression in epithelial HT29 cells. This finding suggested that cell-TNA interaction might not be involved in apoptosis pathway and may allow the cell survival activity. However, expression of p27 and RB proteins indicates the activation of tumor suppressive mechanism on TNA-cells. This mechanism is vital for the cellular balance response between cell proliferation, senescent, and cell death in order to rescue the cell from genotoxicity risk [65]. Thus, cell-TNA interaction might regulate the proliferative activities and respond to positive growth regulation via several genes and protein expressions as illustrated in Figure 6.

As highlighted in this study, cell-TNA interaction might involve the molecular links in the chain of cell homeostasis and redox regulation from GADD45 stress sensor signal that is present at the G0/G1 cell cycle arrest. This interaction could possibly involve the growth regulator team involved in circadian regulators such as CHEK, p53, and MYC. Interestingly, cell-TNA interaction might also trigger cellular sensitivity on DNA repair mechanism via XRCC5 and RAD50 expression as a protective response to offset the impact of the oxidative stress activities and genotoxicity risk. The present study highlights the nanomolecular interaction of epithelial HT29 cell with TNA surface possibly through NF-kappaB/AKT and Wnt signaling. Regulation of these pathways is responsible for cell fate decision via homeostatic proliferation response.

## 5. Conclusion

Epithelial HT29 cells on titania nanotube arrays surface showed an interesting involvement in cellular homeostasis and antioxidant pathways. Discovery and exploitation on innovative nanomaterial products targeted in cellular homeostasis and antioxidant pathways allow better understanding of next generation nanomedicine especially for therapeutic application.

## Figures and Tables

**Table 1:** The target genes for real-time PCR analysis. The gene information was obtained from the website of National Centre of Biotechnology Information (NCBI). The gene sequence primers were selected from TaqMan Assay by Life Technologies, Applied Biosystems (2012) based on the reference sequence number and correct assay ID.

Number	Genes symbol	Ref. Seq	Assay ID
1	18S	X03205	HS99999901_s1
2	EGFR	NM_201282.1	Hs01076078_m1
3	HNRNPA2B1	NM_031243.2	Hs00242600_m1
4	CHEK1	NM_001114121.1	Hs00967506_m1
5	CHEK2	NM_001005735.1	Hs00200485_m1
6	TP53	NM_001126112.1	Hs01034249_m1
7	MYC	NM_002467.4	Hs00153408_m1
8	GADD45A	NM_001199741.1	Hs00169255_m1
9	GADD45G	NM_006705.3	Hs00198672_m1
10	RAD50	NM_005732.3	Hs00990023_m1
11	DDB2	NM_000107.2	Hs03044953_m1
12	XRCC5	NM_021141.3	Hs00221707_m1
13	XRCC6	NM_001469.3	Hs01922652_g1
14	ATM	NM_000051.3	Hs01112307_m1
15	THPO	NM_001177597.1	Hs01061346_m1
16	ANGPTL6	NM_031917.2	Hs00259098_m1
17	MRE11A	NM_005590.3	Hs00967437_m1
